# Response to ‘Major errors in published Salmonella risk assessment model’

**DOI:** 10.1017/S0950268822000802

**Published:** 2022-05-05

**Authors:** F. Sampedro, C. W. Hedberg

**Affiliations:** Environmental Health Sciences, School of Public Health, University of Minnesota, Minneapolis, USA

Dear Editor,

We thank Drs. Zagmutt and Pouillot for their letter identifying some modelling errors related to the D-R equations. We believe that correcting those errors will improve the overall accuracy of the model estimates and give us an opportunity to update the model with new data and insights.

We also wish to state that while the identified errors affect overall estimates of illnesses associated with ground turkey, they do not materially affect our estimates of the relative effect of the interventions we evaluated.

We wish to withdraw our original paper in favour of resubmitting a revised version.

Sincerely,

F. Sampedro and CW Hedberg

